# Phenotypic Quantitative Divergence Across Heterogeneous Environments in a Widespread Southern South American Tree

**DOI:** 10.3390/plants15040618

**Published:** 2026-02-15

**Authors:** Carolina L. Pometti, Juan C. Vilardi, Cecilia F. Bessega

**Affiliations:** Laboratorio de Genética, Facultad de Ciencias Exactas y Naturales, Departamento de Ecología, Genética y Evolución (EGE), Instituto de Ecología, Genética Y Evolución de Buenos de Aires (IEGEBA), Universidad de Buenos Aires (UBA), Consejo Nacional de Investigaciones Científicas y Técnicas (CONICET), Buenos Aires C1428EGA, Argentina; vilardi@ege.fcen.uba.ar (J.C.V.); cecib@ege.fcen.uba.ar (C.F.B.)

**Keywords:** ecological breadth, phenotypic traits, genetic differentiation, local adaptation, *Vachellia caven*, varieties

## Abstract

Phenotypic and genetic divergence along environmental gradients often reflects local adaptation in broadly distributed species. The Fabaceae family is one of the largest and most ecologically important angiosperm groups; it has a centre of diversity in South America and shows high versatility in arid and disturbed environments. Here, we selected *Vachellia* *caven*, a native tree with ecological breadth and taxonomic complexity, to investigate whether phenotypic trait variation among populations reflects adaptive divergence. We examined neutral genetic differentiation in six varieties among populations from Argentina, quantified the phenotypic differentiation of quantitative traits by an ANOVA, and performed *P_ST_—F_ST_* comparisons. We also assessed correlations between phenotypic variation, environmental variables, genotypic variation, and geographic distances. *F_ST_* estimates revealed significant genetic divergence (0.329), in line with isolation by distance and environmental heterogeneity. *P_ST_—F_ST_* comparisons showed that all traits were under diversifying selection, supporting the hypothesis of adaptive phenotypic variation. We further detected that fruit width and length were significantly correlated with specific environmental variables like precipitation and temperature. These findings confirm that phenotypic divergence in *V. caven* is shaped by both geographic and environmental factors. This study offers a preliminary insight into the local adaptation of the examined traits, highlighting how morphological and genetic differentiation has enabled *V. caven* to thrive in diverse environments and contributing information as to how to face climate change scenarios.

## 1. Introduction

Climate change may significantly impact species by altering their geographic distribution, reducing their local populations, and diminishing their genetic diversity, ultimately leading to the ongoing loss of biodiversity [1]. In order to survive, trees need to develop strategies to respond to and cope with recurring stress conditions [2]. The ability of trees to survive through their phenotypic plasticity or potential adaptation is a fundamental issue [3].

Environmental factors are among the main drivers of morphological variation, which results from a combination of phenotypic plasticity and adaptive evolutionary processes. Phenotypic plasticity involves individual acclimation mediated by physiological responses, whereas adaptation is a population-level process involving genetic changes that usually require many generations [4,5].

Phenotypic and genetic divergence observed along environmental gradients or between distinct habitat types often reflects local adaptation in broadly distributed species. Such ecological specialisation may drive speciation processes, and it plays a key role in how populations respond to environmental change [6]. Similarly, tree species that occur across diverse environmental settings are considered to possess a broad ecological niche. Niche breadth refers to the spectrum of environmental conditions encompassed within a species’ realised niche [7]. However, some models assessing the impacts of climate change on tree species tend to overlook niche breadth and intraspecific variation in functional traits under environmental stress [8], potentially leading to significant uncertainty in predicting forest responses to anthropogenic global warming [9]. In this context, we can expect phenotypic trait variation to be adaptive in species with large geographic ranges and possibly also with wide ecological niches. For example, widespread woody species such as *Populus nigra* L., *Eucalyptus risdonii* Hook.f., and *Pinus halepensis* M. Bieb. showed significant variation in traits along climatic gradients, including leaf morphology, growth rates, and phenology, which are linked to adaptive responses [3,10,11].

The Fabaceae family, one of the largest and most ecologically important angiosperm families in the world [12], exhibits high ecological versatility and adaptive capacity in arid and disturbed environments. Species belonging to this family inhabit tropical regions, seasonally dry tropical biomes, and temperate and subtropical zones of the world, where selection pressures vary spatially and temporally [13]. South America is the dominant source of Fabaceae diversity, followed by Africa and temperate Asia [13]. In those environments, morphological differentiation may arise from plastic responses or evolutionary processes driven by selection [14,15]. This is the case of the invasive species *Gleditsia triacanthos* L., a leguminous tree native to US deciduous forests that was found to exhibit local adaptation in seed germination traits and plastic changes in seedling allometry across biomes [16]. Another example is *Neltuma chilensis* (Molina) C.E. Hughes & G.P. Lewis, a tree native to South America; this species showed variation in phenotypic leaf traits in the wild, which would be explained merely by a plastic response to varying environments [17]. In other related leguminous trees also native to South America, like *Neltuma alba* (Griseb.) C.E. Hughes & G.P. Lewis, diversifying selection was found in life history and leaf morphological traits, probably related to heat tolerance and physiological responses [18]. A comparable pattern was reported for *Acacia aroma* Gillies ex Hook. & Arn. (syn. *Vachellia aroma* (Gillies ex Hook. & Arn.) Seigler & Ebinger); the life history traits of this species showed local adaptation and a strong correlation with environmental variables, reflecting the level of perturbation among localities [19]. Overall, this framework helps to separate the different roles of neutral processes and adaptive divergence, particularly in species occurring across environmental gradients.

**Figure 1:**
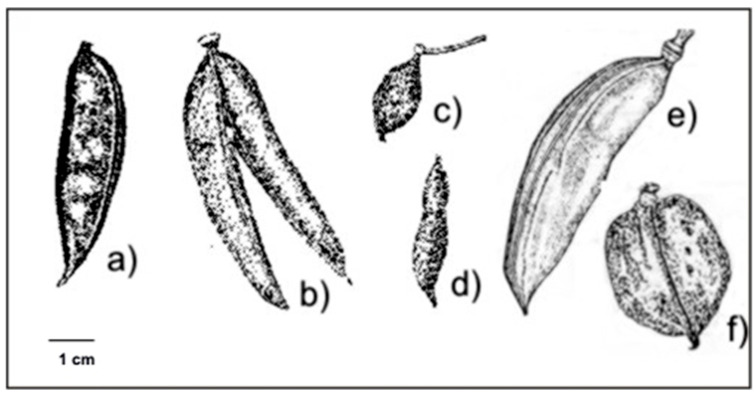
Fruits of the six varieties of *Vachellia caven*. (**a**) *V. c.* var. *caven*, (**b**) *V. c.* var. *dehiscens*, (**c**) *V. c.* var. *microcarpa*, (**d**) *V. c.* var. *stenocarpa*, (**e**) *V. c.* var. *macrocarpa*, (**f**) *V. c.* var. *sphaerocarpa*. Taken from Aronson (1992).

*Vachellia caven* (Molina) Seigler & Ebinger (syn. *Acacia caven* (Molina) Molina) is a tree species native to South America, belonging to the Fabaceae family; it has a broad range of ecological presence and a wide ecological niche [20]. Six varieties were described for this species based on fruit traits [21]: *V. c.* var. *caven*, *V. c.* var. *dehiscens*, *V. c*. var. *sphaerocarpa*, *V. c.* var. *stenocarpa*, *V. c.* var. *microcarpa*, and *V. c. var. macrocarpa* (Figure 1). It should be noted that these varieties are currently regarded as synonyms of *Vachellia caven* by Seigler & Ebinger [22,23], who consider variation in pod morphology to be too high to distinguish varieties. *V. caven* occurs in six countries and can be found in various environments, including temperate grasslands and drylands, where it is most abundant [24]. Argentina is the only country where all six varieties are present; they are distributed across several ecoregions defined by temperature and precipitation gradients [21,25]. This ecological breadth and taxonomic complexity make *V. caven* an ideal model to investigate whether phenotypic trait variation among populations reflects adaptive divergence.

A robust approach to the detection of selective processes in the divergence of quantitative phenotypic traits among populations is based on the comparison of differentiation metrics estimated from quantitative traits, such as *P_ST_* [26,27], with its analogous metric estimated from neutral molecular markers, such as the *F_ST_* [28].

Then, *P_ST_* for a neutrally evolving quantitative trait is expected to be equal to *F_ST_* (a metric that quantifies the average molecular marker differentiation for neutral genetic loci). If *P_ST_* > *F_ST_*, trait divergence exceeds neutral expectation and is likely to have been caused by directional selection. If *P_ST_* < *F_ST_*, trait divergence among populations is less than expected through genetic drift alone; this pattern suggests uniform selection or stabilising selection across populations [29].

Previous works detected genetic and morphological differences among populations belonging to the six varieties of *Vachellia caven* [30,31], advancing the hypothesis that some or all phenotypic trait variation is adaptive. In the present work, we investigate this hypothesis by (1) estimating the neutral genetic differentiation for neutral AFLP loci across 11 populations from five ecoregions of Argentina, representing the six varieties of *Vachellia caven*; (2) measuring the phenotypic differentiation of seven quantitative traits across these populations in order to detect differences among them and to perform the *P_ST_* -*F_ST_* comparisons; and (3) assessing the correlations between phenotypic variation, environmental variables, genetic differentiation and geographic distances.

By integrating neutral genetic markers and environmental and geographic data, we aim to evaluate the extent to which trait variation is shaped by local adaptation; our results will contribute to increasing the insights into evolutionary responses in widespread tree species. We provide robust evidence supporting the hypothesis that, in *V. caven*, phenotypic differentiation exceeds expectations under neutrality, suggesting a role for local adaptation.

## 2. Results

### 2.1. Genetic Characterisation of Vachellia caven

In the analysis for the presence of outliers, 5 of 225 loci appear to be under selection at a 99.5% threshold; therefore, only the 220 neutral loci were considered for further analyses.

The analysis of population structure performed with the software AFLPsurv indicates that the component of variability is higher within populations (*Hw* = 0.275) than among populations (*Hb* = 0.135). The non-hierarchical *F_ST_* is high, 0.329, and highly significant (*p* < 0.001), indicating the presence of genetic structure among populations. Pairwise comparisons of *F_ST_* ranged from 0.050 between PA and VA to 0.500 between TO and FS (Table 1).

### 2.2. Phenotypic Variation

The analysis of the basic statistics for the phenotypic traits across populations show that means for RAL range from 1.665 cm in GY to 3.160 cm in CS; for PLB, from 10 in VA and PA to 18.7 in YP; for PLA, from 12 in VA to 20.6 in YP; for SSL, from 0.403 cm in IB to 2.363 cm in TO; for FPL, from 0.8 cm in VA to 1.569 cm in CS; for FRL, from 2.87 cm in YP to 7.613 cm in TO; and finally, for FRW, from 0.714 cm in FS to 2.169 cm in IB (Table 2). These values, mainly the fruit-related ones, are consistent with the classification into varieties, since YP and FS belong to var. *stenocarpa* (one of the smallest varieties together with *microcarpa*), and both populations present the lowest means in FRL and FRW. Similarly, TO presents the highest mean in FRL since it belongs to the largest variety, *macrocarpa*, and IB presents the highest mean in FRW, consistently with the variety it belongs to, *sphaerocarpa*, the variety with the greatest width.

#### 2.2.1. Analysis of Variance of Morphological Traits

**Figure 2:**
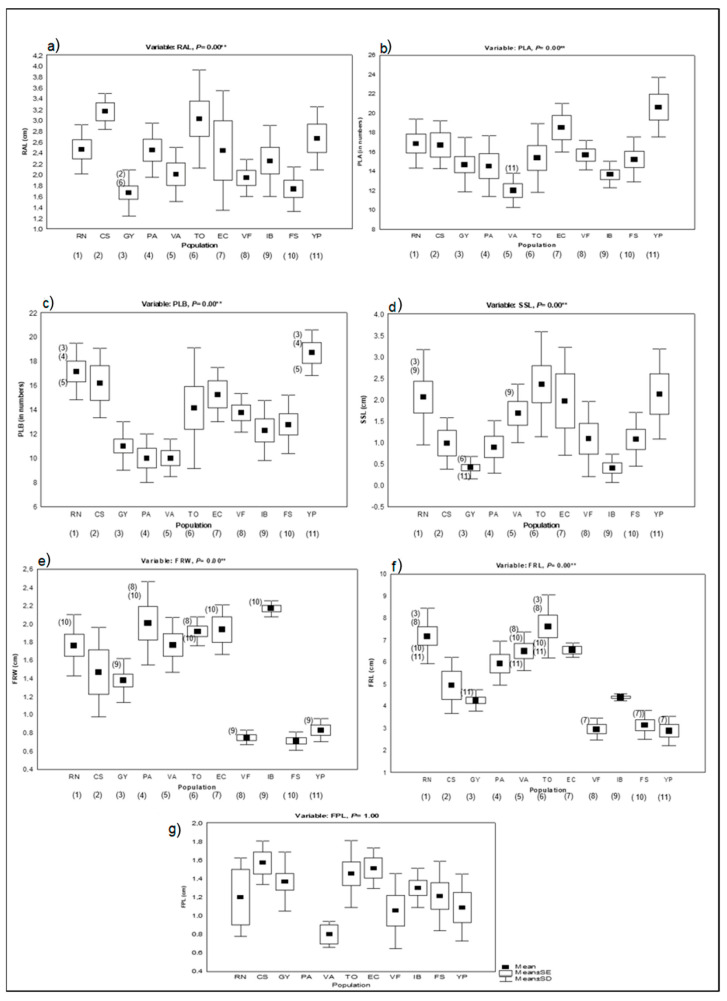
Box-plot showing the comparisons of phenotypic traits measured in 11 populations belonging to the six varieties of *Vachellia caven*. The codes for populations and their corresponding variety are the same as those used in Table 1 and Figure 1. (**a**) RAL, (**b**) PLA, (**c**) PLB, (**d**) SSL, (**e**) FRW, (**f**) FRL, and (**g**) FPL. Statistical significance at the 0.05 level for non-parametric ANOVA. Full squares represent the mean of the trait for each population, empty squares represent the mean ± SD, and vertical lines represent the mean ± SE. The numbers below the name of populations identify the population; the numbers above/below the mean ± SD indicate that this population differs from the number of populations indicated. ** Indicates highly significant.

The Kruskal–Wallis ANOVA reveals highly significant differences among populations (*p* < 0.003) for all traits but fruit peduncle length (FPL) (Figure 2a–f). Post hoc tests on the significant ANOVA results show that, for all traits, some populations exhibit differences from one another, with fruit traits varying between most of the populations (Figure 2e,f). Overall, for fruit width (FRW), *V. c. stenocarpa* differs from the varieties *caven, macrocarpa*, *dehiscens* and *sphaerocarpa*; *V. c. sphaerocarpa* differs from the varieties *caven* and *microcarpa*, and *V. c. microcarpa* differs from the varieties *dehiscens* and *macrocarpa* (Figure 2e). For fruit length (FRL), *V. c.* var. *caven* differs from the varieties *microcarpa*, *stenocarpa* and *macrocarpa*; *V. c.* var. *dehiscens* differs from the varieties *microcarpa* and *stenocarpa*; and *V. c. macrocarpa* differs from the varieties *microcarpa* and *stenocarpa* (Figure 2f).

#### 2.2.2. P_ST_-F_ST_ Comparison

**Figure 3:**
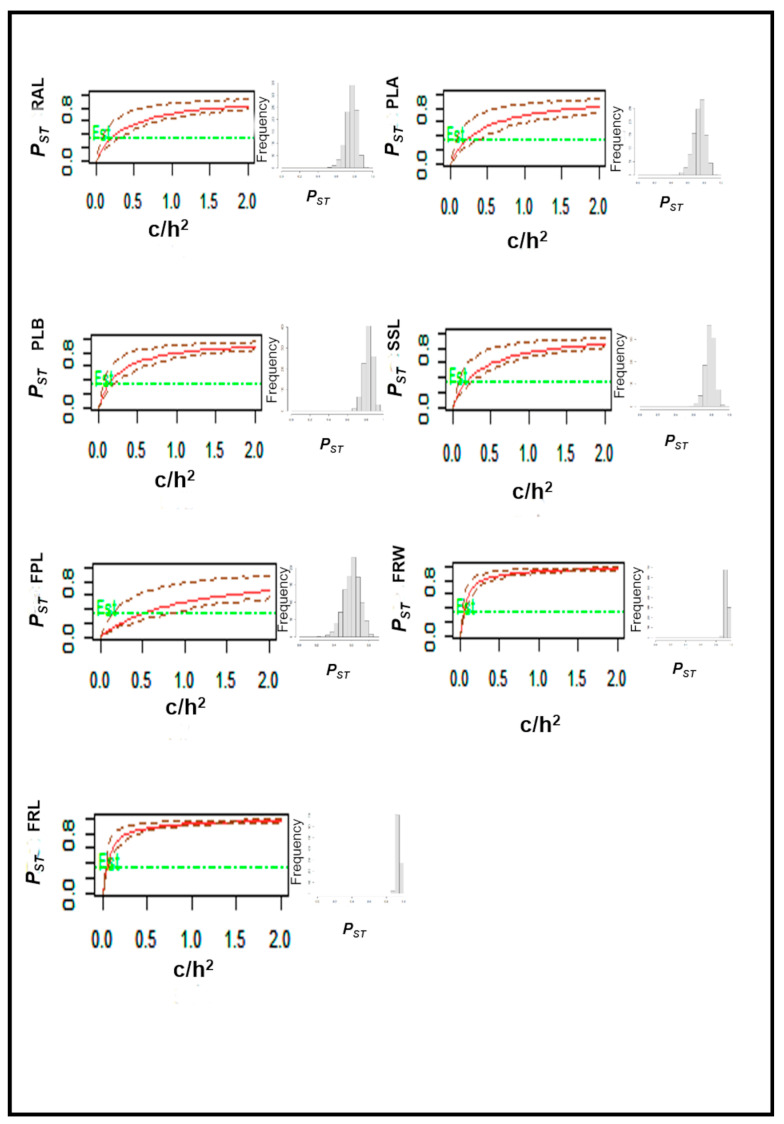
Variation in *P_ST_* for different values of *c/h*^2^ at seven morphological traits. Horizontal green lines represent the *F_ST_* value. Dashed lines represent the 95% confidence interval for *P_ST_* values. The panels on the right show the *P_ST_* distribution histogram of the corresponding evaluated trait.

Phenotypic differentiation (*P_ST_*) was significantly higher than neutral genetic differentiation (*F_ST_*) for all traits, with strong support from local adaptation for a critical c/h^2^ ≤ 0.5 in all traits but FPL (Table 3). For FPL, local adaptation is also suggested, but with a weaker support (c/h^2^ < 1) (Figure 3). The validity of this conclusion was assessed by plotting *P_ST_* and its 95% confidence interval as a function of c/h^2^, overlaid with the neutral expectation, *F_ST_* (Figure 3). Moreover, *P_ST_* distribution histograms confirmed that all traits might be under diversifying selection (skewed distribution, Figure 3).

### 2.3. Environmental and Geographical Variables

The present study involves 11 populations of *Vachellia caven* distributed in five ecoregions with two principal climatic gradients: a precipitation gradient, decreasing from east to west, and a temperature gradient, decreasing from north to south. Considering these environmental variables, together with water vapour pressure (kPa), solar radiation (kJ m^−2^ day^−1^), and wind speed (m s^−1^), and geographic, phenotypic and genetic distances, we evaluated the possible correlations between them and how the environment drives variation in fruit traits.

#### 2.3.1. Correlations Obtained from Mantel and Partial Mantel Tests

We found the existence of isolation by distance (IBD), since the genetic divergence of all the populations was correlated with geographic distances (*p* = 0.003) (Table 4, row 1). Regarding the phenotypic distances, fruit traits were correlated with geographic distances and genetic divergence (FRW, *p* = 0.041 and FRL, *p* = 0.002) (Table 4, rows 2 and 3).

The analysis of the environmental variables with geographic distances and genetic divergence via partial Mantel tests showed that solar radiation (kJ m^−2^ day^−1^), monthly precipitation (mm), monthly mean temperature (°C), monthly max temperature (°C) and wind speed (m s^−1^) are positively and significantly correlated (Table 4, rows 4 to 8). Moreover, the variables monthly precipitation (mm), monthly mean temperature (°C), monthly max temperature (°C), monthly min temperature (°C) and water vapour pressure (kPa) show positive and significant correlations with phenotypic divergence for FRW and FRL and with genetic divergence (Table 4, rows 8 to 17).

#### 2.3.2. Redundancy Analysis (RDA)

Of the seven environmental variables studied, two variables of ecological interest, monthly precipitation (mm) and monthly max temperature (°C), were selected as statistically important, based on forward RDA. These variables explained 91.16% of the variation in fruit traits across populations.

When we corrected the R^2^ for the number of environmental variables in X (the explanatory matrix), the adjusted R^2^ showed that two selected environmental variables explained 67.19% of the variance in the variation in fruit traits. Our full model was statistically significant (*p* = 0.002), and the two environmental variables included in this model were also significant (*p* = 0.01 for Tmax and 0.004 for precipitation). Canonical axis 1 resulting from the RDA was also statistically significant (*p* = 0.001). The projection plot of the fruit traits and the two environmental variables shows that monthly precipitation is a little stronger than monthly max temperature and that the same positive relationship drives the variation in fruit traits (Figure S1).

## 3. Discussion

Species with extensive geographic ranges provide a valuable opportunity to explore how genetic diversity and adaptive responses fluctuate in response to different environmental challenges [32]. This is the first study disentangling the adaptive basis of the six varieties of *Vachellia caven* across heterogeneous environments in Argentina.

The *F_ST_* estimate indicates significant genetic structure, supporting genetic divergence by isolation, which is expected for wide-range species across heterogeneous environments [33]. However, within-population genetic diversity (*H_w_*) exceeds among-population diversity (*H_b_*) because *F_ST_* measures the proportion of total genetic variation that is attributable to differences among populations. Even when most genetic variation is found within populations, a moderate to high *F_ST_* can still emerge if allele frequencies differ consistently across populations [28]. Moreover, in a previous work, we found relatively high expected heterozygosity for *Vachellia caven* with AFLP markers (0.276) [31]; similar estimates were found in other American acacias like *V. curvifructa* (Burkart) Seigler & Ebinger (*H_E_* = 0.21) [34] and *V. aroma* (*H_E_* = 0.21) [35] and African acacias like *Senegalia senegal* Britton (H_E_ = 0.283) [36]. This genetic diversity reflects the evolutionary potential of *V. caven* and its capacity to persist across diverse environments.

Assessing, describing, and analysing morphological variation are key processes for understanding biological adaptability [37]. Moreover, variation in functional traits can be driven by environmental heterogeneity, leading to the emergence of locally adapted ecotypes [38]. In our work, considerable morphological variation was detected by the ANOVA, since it was significant between populations for all traits but fruit peduncle length (FPL), with fruit-related traits showing the most pronounced divergence. Moreover, the *P_ST_-F_ST_* comparison showed that all the studied phenotypic traits were under diversifying selection, with fruit width and length exhibiting the strongest signals. It can be argued that the sample size used for the morphological analyses was relatively small, which may introduce some bias into the results obtained. However, this small sample size was obtained because trait variation was measured using samples from different populations collected in the same year with the aim of minimising the effects of phenotypic plasticity. Despite this, our results indicate that selection, not just genetic drift, is responsible for trait differences, which supports our hypothesis that phenotypic variation in *V. caven* is the result of adaptive processes. These results also validate the assumption that, in tree species with broad geographic ranges and a wide ecological niche, variation in morphological traits could be attributed to local adaptation.

However, due to the nature of *P_ST_-F_ST_* comparisons, phenotypic plasticity could also be inflating *P_ST_* values and cannot be completely disregarded [27,39]. Schmid and Weiner [40] suggested that variability of morphological expressions could be related to habitat types [41]. In *Vachellia nilotica* (L.) P.J.H.Hurter & Mabb, Mahmood et al. [41] found similar results for leaf and stipular spine traits in five populations belonging to different environments and suggested that the differentiation of the phenotypes might result from local variation of the environment; therefore, phenotypic differentiation can be used as an indicator of environmental conditions [41].

The environmental gradients studied (a decreasing E-W precipitation gradient and a decreasing N-S temperature one) may explain the significant differentiation found in leaf, stipular spine and fruit traits. The significant correlation between geographic and environmental distances found here confirms the occurrence of climatic gradients across the sampled populations. Since genetic and environmental distances increase with geographic separation between populations, a greater divergence in phenotypic traits is likely to occur [42,43]. Here, we found that environmental variables like precipitation, temperature and water vapour pressure were significantly correlated with phenotypic variation in fruit traits and genetic divergence, suggesting that climatic factors are key drivers of intra-specific differentiation. More specifically, the results of the redundancy analysis confirmed that precipitation and temperature are positive drivers of the variation in fruit traits. Moreover, fruit traits play a central role in mediating interactions between plants and their animal dispersers and have evolved to match the dispersers’ sensory abilities and morphological characteristics. Simultaneously, these traits are shaped by local environmental conditions, which could influence the degree of trait matching between plants and frugivores. In particular, temperature is a key driver of fruit development, regulating metabolic activity and hormone synthesis, and this fact raises concern in light of the ongoing global warming [44]. These authors claim that other environmental variables play a selective role in climate change; for instance, rainfall patterns determine water availability, which is essential for maintaining cell turgor and allowing pericarp expansion.

Many phylogenetically close Fabaceae species exhibit a quite similar selection pattern under heterogeneous climatic conditions. A study involving eight populations of *Neltuma alba*, for example, showed evidence of local adaptation in three life history traits and seven foliar traits, correlated with differences in temperature, precipitation, wind speed and sunshine fraction [18]. Similar patterns were documented in five populations of *V. nilotica* in Pakistan [41], where local adaptation in leaf and stipular spine traits was detected. The authors attributed these differences to the variation in water availability among populations. A similar situation was described for stipular spines in two South African populations of the widespread species *Vachellia karroo* (Hayne) Banfi & Galasso [45]. Research on *Vachellia aroma*, a species closely related to *Vachellia caven*, showed local adaptation for tree height and a strong correlation with environmental variables, while the remaining 11 traits (leaf, fruit, stipular spine and life history traits) showed evidence of stabilising selection [19]. These examples of closely related wide-range species showed a general trend, in which there is always a selective process acting in response to environmental variables, more precisely, to temperature and precipitation (or water availability). Moreover, in all the cited works, at least some of the traits studied exhibited local adaptation.

The results of the present work also validate the taxonomic classification proposed by Aronson [21] for the six varieties of *V. caven* based mainly on fruit size and shape. Strong adaptive signals in fruit traits support their use in varietal delimitation and show their ecological relevance. Furthermore, the clear match between certain varieties and ecoregions, like *V. c.* var. *dehiscens* with Dry Chaco, *V. c.* var. *sphaerocarpa* with Espinal, *V. c.* var. *macrocarpa* with Puna, *V. c.* var. *microcarpa* with Wet Chaco, and *V. c.* var. *stenocarpa* with Wet Chaco, suggests that ecological differentiation has played a central role in shaping varietal boundaries [31], with no records of populations with intermediate forms or hybrids between varieties [31]. For example, in the Wet Chaco, there is a continuous forest of *V. caven* that shifts from *V. c.* var. *microcarpa* to *V. c.* var. *stenocarpa*, with no individuals exhibiting intermediate forms. Similar results were also found in two varieties of Douglas-fir (*Pseudotsuga menziesii* [Mirb.] Franco) belonging to two contrasting habitats (coastal and interior habitats), where candidate loci associated with local adaptation were identified for both varieties [46]. Such similarities highlight the importance of integrating genetic, morphological, and environmental data to understand intraspecific diversity and its evolutionary drivers.

Climate change has the potential to disrupt local adaptation patterns [1], making it essential to understand genetic diversity and adaptive responses within species, particularly across climatic gradients, to guide effective management, conservation, and restoration strategies [47]. South American ecosystems are undergoing varying degrees of deforestation, mainly due to the increasing global demand for food and the consequent agricultural expansion [48]. Restoring local populations of wide-ranging species and subsequently selecting potential sources for population reintroduction or reinforcement may be difficult. Therefore, conserving the species’ genetic diversity is fundamental since it is associated with evolutionary potential and viability, as well as with the species’ ability to adapt to local and global environmental changes [49]. Based on the results of this study, a sound restoration strategy should involve extensive seed sampling from a broad array of individuals of each population and covering the species’ entire ecological spectrum. From the conservation perspective, including all six taxonomic varieties is essential to maintain genetic variability. In this study, we sampled nearly two populations per variety, which is not enough to provide specific recommendations on the number of populations to preserve per variety. However, individuals from different populations should be preserved since they are locally adapted and would respond, in the long term, to different environmental conditions.

This study represents an initial step toward identifying local adaptation across the traits analysed in the six varieties of *V. caven*, showing that the differences between the *P_ST_* and *F_ST_* provide indications of diversifying selection, especially when trait variation reflects environmental differences among populations. As suggested by Brommer et al. [29], one strategy to identify traits that show signatures of selection may be the application of the *P_ST_*—*F_ST_* comparison; hence, the authors considered them as potentially interesting candidates for studying molecular genetic divergence. The results of the present work improve the knowledge about how natural selection acts in the genus *Vachellia* in South America, where studies sampling a wide portion of the area of distribution of the species are scarce or basically null.

## 4. Materials and Methods

**Figure 4:**
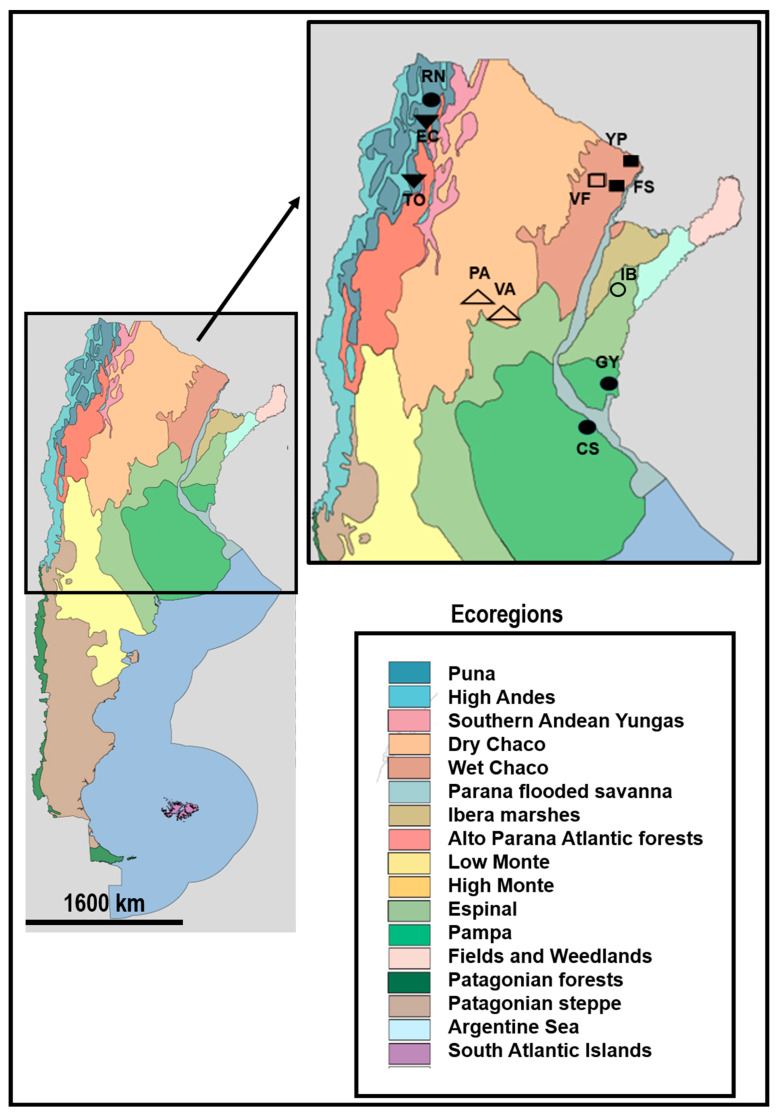
Map of Argentina showing the ecoregions and the amplification of the zone where the 11 populations of *Vachellia caven* were sampled from. Full circles represent *V. c*. var. *caven*; empty triangles represent *V. c.* var. *dehiscens*; full triangles represent *V. c.* var. *macrocarpa*; empty squares represent *V. c.* var. *microcarpa*; empty circles represent * V. c.* var. *sphaerocarpa* and full squares represent *V*. *c*. var. *stenocarpa*. Population abbreviations: CS: Costanera Sur; EC: El Carril; FS: Formosa; GY: Gualeguaychú; IB: Iberá; PA: Pan de Azúcar; RN: Ruta Nueve; TO: Tolombón; VA: Vaquerías; VF: Vivero Forestal.

We studied 11 populations belonging to the six varieties of *Vachellia caven* from five ecoregions of Argentina (Wet Chaco, Dry Chaco, Espinal, Pampa and Puna) (Figure 4; Table 5). Between 11 and 22 individuals were sampled per site, grouping adult trees (trees were considered adults when bearing fruits) and seedlings at the cotyledon stage that were grown in a germination chamber in the lab, totalling 158 individuals.

Representative vouchers of each population were herborised and deposited at the SI herbarium, Instituto de Botánica Darwinion, San Isidro, Buenos Aires, Argentina.

### 4.1. AFLP Methods and Data Analysis

For adults, young, fully grown, healthy leaves were collected and kept in bags with silica gel until DNA extraction. For seedlings, cotyledons were ground to a fine powder in liquid nitrogen and then placed in a microtube. The DNeasy Plant kit (QIAGEN Inc., Valencia, California, USA) was used for the DNA extraction of the total number of individuals, following the manufacturer’s instructions. DNA was stored at −20 °C.

The AFLP technique was performed as described by Vos et al. [50], following the steps detailed in Pometti et al. [31]. Some samples used here have featured in a previous study of landscape genetics [31].

The AFLP dataset consisted of 225 putatively neutral loci (the presence of outliers was checked with the software BAYESCAN v2.1; [51]) of 158 individuals of *V. caven* from 11 populations belonging to the five previously mentioned ecoregions.

Each AFLP band was coded for presence (1) or absence (0). Allele frequencies were estimated with the software AFLP-SURV 1.0 [52], using the Bayesian method with a non-uniform prior distribution of allele frequencies, as described by Zhivotovsky [53], following Lynch and Milligan’s [54] approach. Non-hierarchical Wright’s [55] *F_ST_*, pairwise *F_ST_* between populations and population genetic structure (*Hw*, *Hb* and *H_T_*) were also estimated using the software AFLP-SURV [50]. Statistical significance for *F_ST_* was determined using 1000 permutations.

### 4.2. Phenotypic Variation

Seven morphological traits related to fruit and leaf morphology, and stipular spines, were measured in 74 adult individuals. These traits can be used to measure the impact of environmental factors on plant physiology, since they are typically associated with plant responses to environmental stress and are relatively simple to measure [56]. The traits related to fruit morphology were selected due to their importance in the classification of the six varieties of *V. caven* and their taxonomic importance in the genus *Vachellia*. Moreover, fruit traits are fitness-related, since they are responsible for seed dispersal and nutrient acquisition by frugivores [57]. Functional leaf traits are linked to structural attributes and carbon assimilation and the capacity to cope with environmental conditions [58,59]. Furthermore, these traits contribute to mechanical resilience under challenging conditions, including elevated temperatures, strong winds, and water scarcity [60]. Stipular spines are a defence strategy against herbivory in *Vachellia*. According to Coley et al. [61], under resource-limited conditions, plants with inherently slow growth rates are favoured over fast-growing species because slower growth tends to be associated with greater investment in defence mechanisms. The leaf and stipular spine traits chosen here have shown local adaptation in other leguminous species [18,39,43].

**Figure 5:**
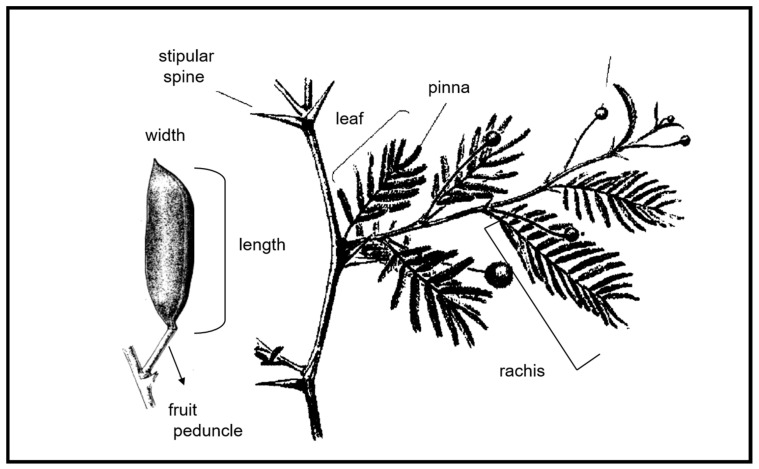
Morphological traits measured in *Vachellia caven*.

The traits measured were rachis length (cm) (RAL), the number of pairs of leaflets on the apical pinna (PLA), the number of pairs of leaflets on the basal pinna (PLB), stipular spine length (cm) (SSL), fruit peduncle length (cm) (FPL), fruit length (cm) (FRL), and fruit width (cm) (FRW) (Figure 5).

Measurements were taken on three replicates of the herbarium material collected at varying heights of the tree, and statistics were based on the average of these measurements for each individual. All measurements were taken by the same researcher (CP) to the nearest millimetre with a hand ruler. The original data matrix is available from the authors upon request.

To assess the occurrence of significant differences among populations for each trait, a nonparametric Kruskal–Wallis [62] ANOVA was applied. We chose this test because it is a distribution-free method, i.e., it does not require any assumption about trait distribution. When ANOVA was significant, Dunn’s post hoc tests for multiple comparisons were performed for each trait to estimate pairwise differences between populations. These analyses were conducted using the software Statistica 7.0 [63].

To assess the role of natural selection in *Vachellia caven*, quantitative traits and genetic differentiation were compared among populations (*P_ST_*—*F_ST_* comparison). As stated by Leinonen et al. [27] and Brommer [26], *P_ST_* can be considered a valid alternative when *Q_ST_* estimates are unavailable.

*P_ST_* was calculated following the equation proposed by Brommer [26]:

$$P_{\mathrm{ST}}=\frac{\left(c/h^{2}\right){\sigma_{B}}^{2}}{\left(c/h^{2}\right){\sigma_{B}}^{2}+2\left({\sigma_{W}}^{2}\right)}$$

where *σ_B_*^2^ is the phenotypic variance between groups (populations), *σ_W_*^2^ is the phenotypic variance within groups, and the *c/h*^2^ ratio is the proportion of additive variance across populations relative to the within-population heritability.

*P_ST_* values for each trait were calculated with the Pstat package version 1.2 [64] in R software 4.5.1 [65], as their confidence intervals (upper and lower 95% CI) with 9999 bootstraps and distribution histograms. In addition, variations of *P_ST_* for each trait as a function of c/h^2^ were studied. Local adaptation is likely when *P_ST_* and its 95% confidence interval (upper and lower) exceed *F_ST_* for a critical c/h^2^ < 1, and it is strongly confirmed for a critical c/h^2^ ≤ 0.5 [26]. Additionally, *P_ST_* histograms with normal distribution indicate that the evaluated trait (or traits) does not show evidence of selection.

### 4.3. Environmental and Geographical Variables

In order to check the possible correlations between *P_ST_* pairwise, *F_ST_* pairwise, geographic distances, and environmental variables, different Mantel and partial Mantel tests were conducted with the vegan package [66] in R software 4.5.1 [65] with 9999 permutations. We considered seven environmental variables reflecting the differences among populations at 30 s spatial resolution (~1 km) obtained from the WorldClim 2.1 [67] database, averaged for the 1970–2000 period. The variables selected for each population were: monthly mean temperature (°C), monthly max temperature (°C), monthly min temperature (°C), monthly precipitation (mm), water vapour pressure (kPa), solar radiation (kJ m^−2^ day^−1^), and wind speed (m s^–1^). All data were extracted from geotiff files using the R package raster [68]. For each variable, a Euclidean distance matrix was obtained.

Geographic distances between sample sites were estimated using Google Earth Pro 7.3.6.10201 measuring tool (2025) (http://earth.google.es accessed on 28 February 2025).

In order to identify how environmental variables drive the variation in fruit traits (FRW and FRL), a redundancy analysis (RDA) [69] was performed with the package vegan [66] of the R software 4.5.1 [65]. The explanatory matrix X contained the environmental variables of each population, while the response matrix Y contained the fruit traits of each population. All variables were standardised before the RDA was conducted. To reduce the number of environmental variables and, therefore, avoid collinearity and to retain statistically important variables and those of ecological importance, a forward selection was performed before conducting the RDA.

## 5. Conclusions

The morphological and genetic differentiation among *Vachellia caven* populations has enabled the species to persist across diverse environmental conditions and, therefore, to occur in a broad range of ecological niches. This is valuable information in the context of future climate change scenarios. The results obtained here highlight the importance of integrating genetic, morphological, and ecological data to inform taxonomy, conservation, and restoration in wide-ranging species. Regarding the taxonomic classification, we consider that our study validates the six varieties for *V. caven* proposed by Aronson [21], which were ecologically differentiated, as stated previously. The establishment of a common garden assay in the future would allow us to determine whether they are varieties, as we validated morphologically and genetically in previous works [30,31], different species or just ecological types. This is a first step to detecting selection in natural populations of *Vachellia caven*; future studies should include common garden assays or reciprocal transplant experiments to provide further support for our results.

## Figures and Tables

**Table 1:** Pairwise *F_ST_* comparisons among populations of *Vachellia caven.* Abbreviations of populations: CS: Costanera Sur; EC: El Carril; FS: Formosa; GY: Gualeguaychú; IB: Iberá; PA: Pan de Azúcar; RN: Ruta Nueve; TO: Tolombón; VA: Vaquerías; VF: Vivero Forestal.

Population	CS	EC	FS	GY	IB	PA	RN	TO	VA	VF
CS										
EC	0.277									
FS	0.492	0.425								
GY	0.226	0.280	0.376							
IB	0.340	0.321	0.340	0.227						
PA	0.303	0.236	0.351	0.209	0.236					
RN	0.311	0.346	0.447	0.283	0.274	0.279				
TO	0.457	0.351	0.500	0.362	0.418	0.362	0.483			
VA	0.260	0.207	0.370	0.205	0.255	0.050	0.289	0.334		
VF	0.456	0.384	0.111	0.348	0.299	0.320	0.430	0.441	0.331	
YP	0.446	0.395	0.117	0.334	0.264	0.333	0.395	0.458	0.338	0.084

**Table 2:** Basic statistics of the phenotypic traits: means, standard deviations (in parentheses), N = the sample size of each trait in each studied population of *V. caven*. NA = data not available.

	** Populations**										
**Trait**	RN	CS	GY	PA	VA	TO	EC	VF	IB	FS	YP
**Rachis length (RAL) (cm)**	2.464 (0.393) N = 9	3.160 (0.330) N = 4	1.665 (0.422) N = 12	2.450 (0.502) N = 6	2.008 (0.499) N = 6	3.025 (0.908) N = 8	2.443 (1.102) N = 4	1.942 (0.337) N = 6	2.254 (0.649) N = 7	1.736 (0.410) N = 7	2.670 (0.577) N = 5
**Pairs of leaflets on the basal pinna (PLB) (number)**	17.143 (2.027) N = 9	16.208 (2.860) N = 4	11.000 (2.000) N = 12	10.000 (2.00) N = 6	10.000 (1.549) N = 6	14.125 (4.970) N = 8	15.250 (2.217) N = 4	13.750 (1.605) N = 6	12.287 (2.476) N = 7	12.786 (2.395) N = 7	18.700 (1.891) N = 5
**Pairs of leaflets on the apical pinna (PLA) (number)**	16.857 (2.204) N = 9	16.708 (2.495) N = 4	14.667 (2.774) N = 12	14.500 (3.146) N = 6	12.000 (1.789) N = 6	15.375 (3.583) N = 8	18.500 (2.517) N = 4	15.667 (1.506) N = 6	13.667 (1.389) N = 7	15.214 (2.325) N = 7	20.600 (3.070) N = 5
**Stipular spine length (SSL) (cm)**	2.056 (1.111) N = 9	0.983 (0.602) N = 4	0.413 (0.266) N = 12	0.892 (0.617) N = 6	1.675 (0.684) N = 6	2.363 (1.227) N = 8	1.963 (1.261) N = 4	1.083 (0.877) N = 6	0.403 (0.333) N = 7	1.071 (0.631) N = 7	2.130 (1.052) N = 5
**Fruit peduncle length (FPL) (cm)**	1.200 (0.150) N = 9	1.569 (0.236) N = 4	1.367 (0.317) N = 12	NA	0.800 (0.063) N = 6	1.450 (0.359) N = 8	1.513 (0.217) N = 4	1.053 (0.404) N = 6	1.300 (0.208) N = 7	1.213 (0.374) N = 7	1.090 (0.361) N = 5
**Fruit length (FRL) (cm)**	7.175 (1.166) N = 9	4.939 (1.284) N = 4	4.254 (0.479) N = 12	5.933 (1.005) N = 6	6.492 (0.876) N = 6	7.613 (1.429) N = 8	6.543 (0.334) N = 4	2.952 (0.490) N = 6	4.400 (0.173) N = 7	3.137 (0.656) N = 7	2.870 (0.663) N = 5
**Fruit width (FRW) (cm)**	1.763 (0.316) N = 9	1.469 (0.492) N = 4	1.379 (0.239) N = 12	2.008 (0.458) N = 6	1.767 (0.303) N = 6	1.919 (0.162) N = 8	1.938 (0.275) N = 4	0.748 (0.075) N = 6	2.169 (0.088) N = 7	0.714 (0.100) N = 7	0.830 (0.125) N = 5

**Table 3:** *P_ST_* values for phenotypic traits in *Vachellia caven* and its 95% confidence interval, with *c/h*^2^ = 0.5. Codes for traits: rachis length (cm) (RAL), pairs of leaflets on the apical pinna (PLA), pairs of leaflets on the basal pinna (PLB), stipular spine length (cm) (SSL), fruit peduncle length (cm) (FPL), fruit length (cm) (FRL) and fruit width (cm) (FRW).

Trait	*P_ST_*	95% CI
RAL	0.547	0.475–0.768
PLA	0.525	0.410–0.762
PLB	0.649	0.549–0.823
SSL	0.580	0.512–0.767
FPL	0.329	0.251–0.626
FRW	0.867	0.826–0.938
FRL	0.867	0.822–0.943

**Table 4:** Results of significant Mantel and partial Mantel tests for geographic distances, genetic and phenotypic divergence, and environmental variables.

Mantel/Partial Mantel Test	R	*p*
Geographic distances vs. *F_ST_*	0.444	0.003
Geographic distances vs. FRW vs. *F_ST_*	0.236	0.041
Geographic distances vs. FRL vs. *F_ST_*	0.479	0.002
Geographic distances vs. solar radiation vs. *F_ST_*	0.457	0.001
Geographic distances vs. monthly precipitation vs. *F_ST_*	0.419	0.004
Geographic distances vs. monthly mean temperature vs. *F_ST_*	0.285	0.029
Geographic distances vs. monthly max temperature vs. *F_ST_*	0.500	0.001
Geographic distances vs. wind speed vs. *F_ST_*	0.665	1 × 10^−4^
FRL vs. monthly precipitation vs. *F_ST_*	0.403	0.006
FRW vs. monthly mean temperature vs. *F_ST_*	0.505	0.003
FRL vs. monthly mean temperature vs. *F_ST_*	0.422	0.007
FRW vs. monthly max temperature vs. *F_ST_*	0.420	0.002
FRL vs. monthly max temperature vs. *F_ST_*	0.400	0.009
FRW vs. monthly min temperature vs. *F_ST_*	0.544	7 × 10^−4^
FRL vs. monthly min temperature vs. *F_ST_*	0.460	0.003
FRW vs. water vapour pressure vs. *F_ST_*	0.446	0.004
FRL vs. water vapour pressure vs. *F_ST_*	0.360	0.016

**Table 5:** Names, abbreviations and coordinates of the 11 populations of *Vachellia caven*. Variety and ecoregion corresponding to which each population. N = sample size.

Variety	Ecoregion	Population	Population Code	Latitude (°S)	Longitude (°W)	N
*caven*	Pampa	Costanera Sur	CS	34°38′10.71″	58°42′44.08″	16
*caven*	Pampa	Gualeguaychú	GY	33°22′4.00″	58°44′3.00″	22
*caven*	Puna	Ruta Nueve	RN	24°39′48.00″	65°22′49.00″	14
*macrocarpa*	Puna	El Carril	EC	25° 4′58.80″	65°28′1.20″	14
*macrocarpa*	Puna	Tolombón	TO	26°11′8.00″	65°56′7.00″	14
*microcarpa*	Wet Chaco	Vivero Forestal	VF	26°16′0.00″	58°17′41.64″	12
*stenocarpa*	Wet Chaco	Formosa	FS	26°16′13.20″	58°17′7.92″	12
*stenocarpa*	Wet Chaco	YPF	YP	26°11′26.76″	58° 9′23.82″	14
*sphaerocarpa*	Espinal	Iberá	IB	28°15′40.13″	56°30′20.38″	17
*dehiscens*	Dry Chaco	Pan de Azúcar	PA	31°15′58.90″	64°20′28.60″	11
*dehiscens*	Dry Chaco	Vaquerías	VA	31°23′38.93″	63°51′30.87″	12

## Data Availability

The data underlying this article will be shared upon reasonable request to the corresponding author.

## Supplementary Materials

The following supporting information can be downloaded at https://www.mdpi.com/article/10.3390/plants15040618/s1: Figure S1: Projection of fruit traits (FRW and FRL, in red case), populations (in black case) and environmental variables in the RDA performed on the morphological dataset of *Vachellia caven*. The first two axes of the RDA projection represent 96% (RDA1) and 6% (RDA2) of the explained variance.
