# Supplementary figures and images for: Phenotypic Quantitative Divergence Across Heterogeneous Environments in a Widespread Southern South American Tree
Source: Plants (Basel). 2026 Feb 15;15(4):618. doi: 10.3390/plants15040618 (PMC12944476; doi:10.3390/plants15040618)

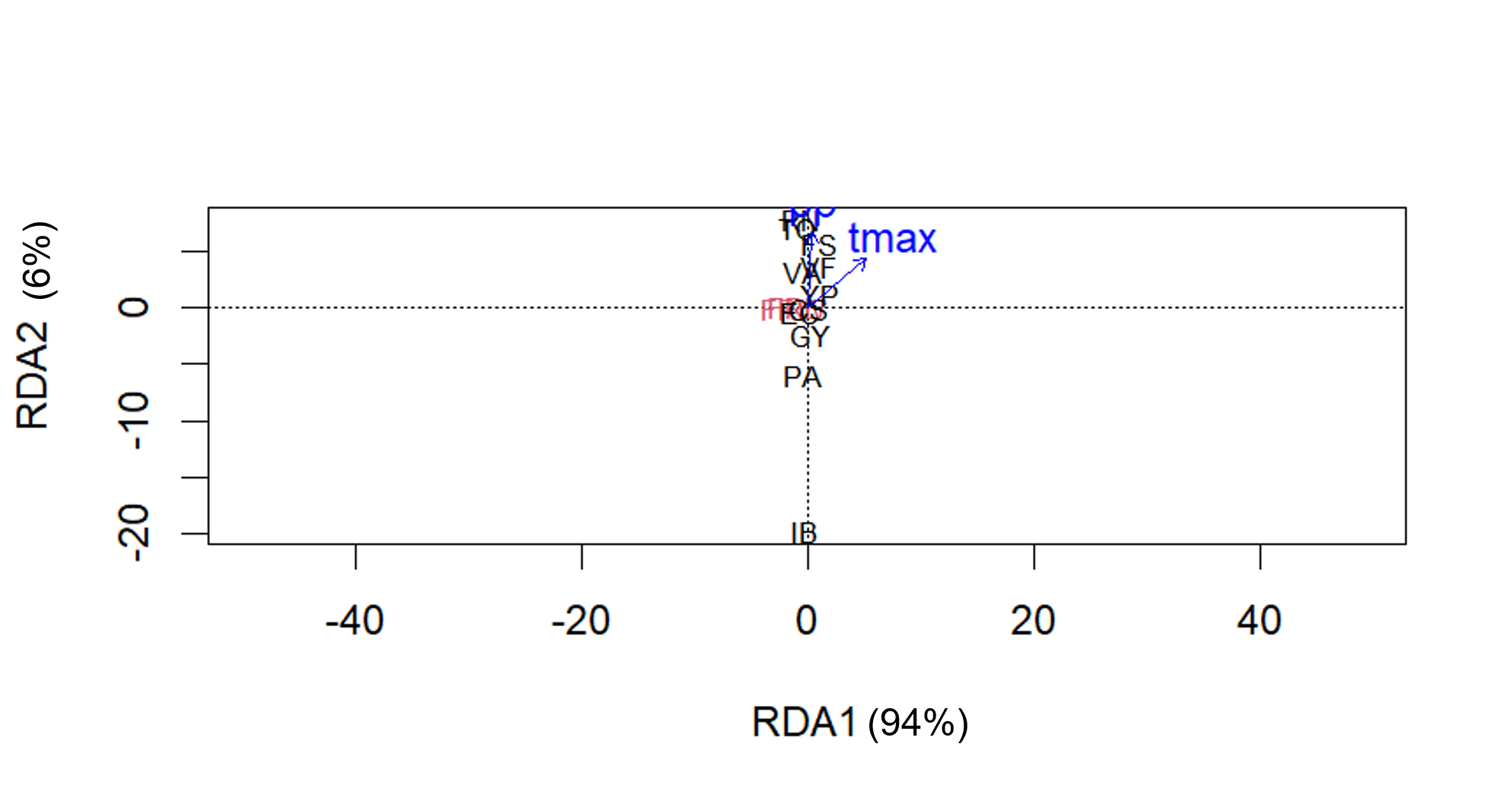

Supplement: Supplementary file 1 [file plants-15-00618-s001.zip › plants-4070233-supplementary.png]
